# When given the opportunity, chimpanzees maximize personal gain rather than “level the playing field”

**DOI:** 10.7717/peerj.165

**Published:** 2013-09-17

**Authors:** Lydia M. Hopper, Susan P. Lambeth, Steven J. Schapiro, Sarah F. Brosnan

**Affiliations:** 1 Lester E. Fisher Center for the Study & Conservation of Apes, Lincoln Park Zoo, USA; 2 Michale E. Keeling Center for Comparative Medicine and Research, UT MD Anderson Cancer Center, Bastrop TX, USA; 3 Language Research Center, Georgia State University, Atlanta GA, USA; 4 Department of Experimental Medicine, University of Copenhagen, Copenhagen, Denmark; 5 Department of Psychology, Philosophy & Neuroscience Institute, Georgia State University, Atlanta GA, USA

**Keywords:** *Pan troglodytes*, Chimpanzee, Response facilitation, Social facilitation, Inequity, Individual contrast, Food choice

## Abstract

We provided chimpanzees (*Pan troglodytes*) with the ability to improve the quality of food rewards they received in a dyadic test of inequity. We were interested to see if this provision influenced their responses and, if so, whether it was mediated by a social partner’s outcomes. We tested eight dyads using an exchange paradigm in which, depending on the condition, the chimpanzees were rewarded with either high-value (a grape) or low-value (a piece of celery) food rewards for each completed exchange. We included four conditions. In the first, “Different” condition, the subject received different, less-preferred, rewards than their partner for each exchange made (a test of inequity). In the “Unavailable” condition, high-value rewards were shown, but not given, to both chimpanzees prior to each exchange and the chimpanzees were rewarded equally with low-value rewards (a test of individual contrast). The final two conditions created equity. In these High-value and Low-value “Same” conditions both chimpanzees received the same food rewards for each exchange. Within each condition, the chimpanzees first completed ten trials in the Baseline Phase, in which the experimenter determined the rewards they received, and then ten trials in the Test Phase. In the Test Phase, the chimpanzees could exchange tokens through the aperture of a small wooden picture frame hung on their cage mesh in order to receive the high-value reward. Thus, in the Test Phase, the chimpanzees were provided with an opportunity to improve the quality of the rewards they received, either absolutely or relative to what their partner received. The chimpanzees responded in a targeted manner; in the Test Phase they attempted to maximize their returns in all conditions in which they had received low-value rewards during the Baseline Phase. Thus, the chimpanzees were apparently motivated to increase their reward regardless of their partners’, but they only used the mechanism provided when it afforded the opportunity for them to increase their rewards. We also found evidence that the chimpanzees’ responses were enhanced by social facilitation. Specifically, the chimpanzees were more likely to exchange their tokens through the frame when their test partner also did so, even in circumstances in which their reward value could not be improved. Our paradigm provided the chimpanzees with the possibility to improve the quality of rewards they received in the Test Phase. We found that refusals – to exchange tokens or to eat rewards – decreased significantly in the Test Phase compared to the Baseline Phase, where no such opportunity for improvement of outcomes existed. Thus, the chimpanzees participated more when they could improve the rewards they received.

## Introduction

Animals’ food choices cannot be evaluated in a vacuum; the selections they make are under a number of social and environmental influences (Galef, 1996). While some foods that animals choose to consume reflect nutritional requirements or those foods that are available in their environment, social pressures (e.g., Murray, Eberly & Pusey, 2006; Hopper et al., 2011) and taste (e.g., Wrangham, Conklin-Brittain & Hunt, 1998; Remis, 2002; Remis, 2006) also impact food choices. Nonhuman primates’ foraging patterns are known to be affected by social influences that dictate when, where, and for what foods they forage (e.g., Nishida, 1968; Alberts, Altmann & Wilson, 1996; Bates & Byrne, 2009). Additionally, primates, and other animals, preferentially choose to eat foods that were previously selected by social partners (e.g., van de Waal, Borgeaud & Whiten, 2013), even if it is not their preferred food (Sherwin, Heyes & Nicol, 2002; Galef & Whiskin, 2008; Hopper et al., 2011). This effect of social facilitation is so strong that monkeys, at least, will consume more if a social partner is eating in their presence, even if the partner is receiving a more preferred food than is the subject (Dindo & de Waal, 2007). This is particularly interesting given the propensity of some primate species to recognize, and respond to, inequitable outcomes for completing a task (i.e., an unequal distribution of rewards, (Brosnan, 2011; Price & Brosnan, 2012) provide reviews).

A potential underpinning of the recognition of inequity is the contrast effect, whereby an individual reacts negatively when an expected, and desired, item is replaced with a less desirable one (Tinklepaugh, 1928). Frustration responses to individual contrast are shown by both humans and nonhuman animals (Reynolds, 1961; Friedan, Cuello & Kacelnik, 2009; Talbot et al., 2011). The key difference between a response to individual contrast and a response to inequity is the form of the comparison; the latter requires social comparison while the former does not require individuals to recognize others’ outcomes. A response to individual contrast emerges when, for example, an individual refuses a poorer quality reward offered for completing a task after having been previously offered a better reward for completing the same task (Roma et al., 2006). In comparison, an inequity response occurs if an individual refuses a poor-quality reward for completing the same task as their partner, who received a more desirable reward (reviewed in Brosnan, 2013).

Typical tests of inequity with nonhuman primates include conditions which create individual contrast and conditions of inequity to tease apart the relative impact of the two (e.g., Brosnan et al., 2010). Furthermore, it has been proposed that individuals only respond to inequity when it results from unequal rewards given after the completion of a task, rather than when rewards are provided to subjects for “free” (e.g., van Wolkenten, Brosnan & de Waal, 2007). Such tests also include conditions in which the animals have to work for their rewards (e.g., exchange a token with an experimenter) and compare their responses to conditions in which they do not work (e.g., get the rewards for free; Talbot et al., 2011). Indeed, this impact of “effort” could explain why primates that dislike inequity (Brosnan & de Waal, 2003) also eat more of a less preferred outcome when their partners are provided better rewards (Dindo & de Waal, 2007).

Given the potentially conflicting influences of social facilitation and inequity on the selection of foods that primates choose to eat, we wanted to test primate food choices in a test of inequity. Thus, we wished to determine if primates attempted to improve the quality of food rewards that they could gain depending on the quality of rewards they received in comparison to their partner’s. Chimpanzees (*Pan troglodytes*), a highly gregarious nonhuman primate that live in large multi-male, multi-female groups maintained by strong and complex social bonds, affiliations and hierarchies (Smuts et al., 1987; Mitani et al., 2012), were selected as the subjects for this study. We tested chimpanzees because of their tendency to attend to the actions and outcomes of their peers’ behavior (Hopper et al., 2007; Hopper et al., 2008, but see Tennie, Call & Tomasello, 2012) and because they are known to respond to social contingencies (Brosnan et al., 2010). Specifically, chimpanzees appear to assess the quality of their food rewards in relation to those of social partners (Horner et al., 2011) and they sometimes reject food offered to them if it is of lesser quality than food offered to their peers (Brosnan et al., 2010, but see Bräuer, Call & Tomasello, 2009). Therefore, we predicted that chimpanzees would likely be sensitive to foods available within their social environment and attempt to adjust the rewards available to them when given the opportunity to do so.

We tested whether chimpanzees would attempt to increase their personal gain in a typical token exchange test of inequity (c.f. Brosnan et al., 2010). Specifically, we provided chimpanzees with the opportunity to exchange tokens through the aperture of small wooden picture frames hung on their cage mesh and, if they took the time and care to do so, they were rewarded with a more desirable food reward. If the chimpanzees took advantage of this mechanism for obtaining a more preferred reward, we wished to ascertain whether this response was to increase their rewards absolutely or relative to their partner’s rewards, and also whether they did so preferentially when doing so was a “trade up” rather than when they were already receiving the more preferred reward. We predicted that the chimpanzees would (i) attempt to procure more desirable food rewards and (ii) engage more in the task when this opportunity to improve their rewards was provided.

## Materials and Methods

### Subjects and housing

We tested 16 captive chimpanzees (eight males, eight females), with an average age of 29.4 years (range: 17–50 years). All chimpanzees were socially housed at the Michale E. Keeling Center for Comparative Medicine and Research, UT MD Anderson Cancer Center, USA, and all had participated in previous comparable studies of social comparison, but without the opportunity to change the rewards they were offered (e.g., Brosnan et al., 2010). UT MD Anderson is fully accredited by the Association for the Assessment and Accreditation of Laboratory Animal Care-International and approval for the chimpanzee study was gained from the Institutional Animal Care and Use Committee (IACUC approval number: 07-92-03887) of UT MD Anderson.

The chimpanzees were tested in eight unique pairs comprised of familiar cagemates (three female–female pairs, three male-male pairs and two male–female pairs). Following Brosnan et al. (2010), each test pair was unique so each chimpanzee was only tested with one partner. All the chimpanzees voluntarily participated in the study. Each day, the experimenter would call the chimpanzees into one of the indoor dens of their enclosure and only those animals that chose to come in for testing participated that day. Each pair of chimpanzees was tested in the same inside den of their enclosure so that they could easily see the actions of their partner and what food reward their partner received. Thus, subjects were sitting next to one another, not across from one another. During tests, these pairs did not have visual access to the rest of their group. Each test lasted no more than 25 min and immediately after a test was completed, the chimpanzees returned to their social group. At all other times, the chimpanzees had access to large, highly enriched indoor/outdoor enclosures. During test sessions (and at all other times), chimpanzees had *ad libitum* access to water and primate chow. Outside of testing periods, the chimpanzees also received three meals of fresh produce (fruit and vegetables) daily.

### Food preference testing

Prior to running any session, to determine food items for the high-value and low-value rewards, we ran a series of dichotomous forced-choice tests with all of the chimpanzees. For these, chimpanzees were individually offered a choice between two food items. The experimenter held a piece of each of the two foods, with one held in one hand, and the other held in her other hand, and then presented her outstretched hands simultaneously to the chimpanzee. Once the chimpanzee selected one (by reaching for it with either his/her hand or mouth) s/he was given it to eat and the other food option was withdrawn. In this way, the chimpanzees could only obtain one of the two offered foods. This same dichotomous choice was presented to the chimpanzees on 10 trials on one day and a further 10 trials on a second day. For each trial, the side of each food presentation was alternated to avoid food choices being conflated with side preferences. These tests were run with every chimpanzee until one food was found to be consistently selected by all 16 chimpanzees over the other in at least 8 out of 10 trials on both days. Thus, we did not exclude chimpanzees that did not prefer a particular food, but selected foods as a reflection of the choices that the chimpanzees made. These tests determined that the universal high-value reward was a grape (as has been used with these chimpanzees in previous tests of inequity and social learning, e.g., Brosnan et al., 2010; Hopper et al., 2011). The chimpanzees universally selected grapes over a grape-sized piece of celery, but were still willing to consume celery when grapes were not available, and so celery pieces were then assigned as the low-value reward.

### Experimental design

The chimpanzees were tested in unique pairings in a series of four conditions (Table 1). Within each test session, one chimpanzee acted as the ‘subject’ and one as the ‘partner’. Chimpanzees were tested in both roles and were tested twice in each role in each condition. All conditions were administered in a counterbalanced manner. Following Brosnan et al. (2010), in each condition, chimpanzees took turns exchanging a PVC token (a piece of pipe that was 20 cm long and 5 cm in diameter) with the experimenter and were rewarded with a piece of food for each completed exchange. All chimpanzees were already familiar with exchanging tokens with an experimenter and so no training for this was required.

**Table 1:** The experimental conditions. In each condition, the chimpanzees were given a food reward for each completed exchange. Depending on the condition these rewards were either of equal or different value to that offered to their test partner. In the Unavailable condition only, prior to making an exchange, the chimpanzees were shown a high-value reward, but upon completion of their exchange, they were given a low-value reward. In the Test Phase, if the chimpanzees exchanged a token through the picture frame then they would receive a high-value reward (a grape), irrespective of the condition. Thus, the subjects had the ability, in the Test Phase, to gain a more preferred rewards in the Different, Unavailable and Low-value Same conditions. In the High-value Same condition, because the chimpanzees were already receiving the high-value reward, exchanging tokens through the picture frame would not impact the reward they would be given.

Condition	Subject		Partner	
	Shown	Given	Shown	Given
Different	—	Celery	—	**GRAPE**
Unavailable	**GRAPE**	Celery	**GRAPE**	Celery
Low-value Same	—	Celery	—	Celery
High-value Same	—	**GRAPE**	—	**GRAPE**

**Figure 1:**
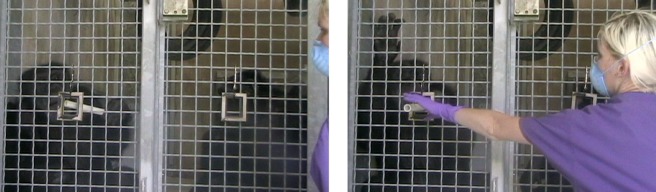
Exchanging tokens through the picture frames in the Test Phase. Stills from video footage of the Test Phase showing the Subject chimpanzee returning his token through his picture frame. As shown, to avoid cuing the chimpanzees’ responses, the experimenter only reached for the token when the chimpanzee had pushed 50% of the token through the picture frame. Note, although there is a central support bar in the middle of the mesh, the chimpanzees are in the same enclosure and have full visual access to both food rewards outside the cage and also to the actions of their test partner.

Each experimental condition had two Phases (Baseline and Test) that were run consecutively. In the Baseline Phase, starting with the partner, the two chimpanzees took turns exchanging for 20 total trials (10 exchanges per chimpanzee). Depending on the condition, for every completed exchange, the subject either received the same reward or a different reward as compared to their test partner (Table 1). The foods were classed as either more preferred, high-value rewards (a grape) or less-preferred, low-value rewards (a piece of celery). After each chimpanzee had been given the opportunity to make ten exchanges, they were then tested in the Test Phase. In the Test Phase, the subjects could improve the quality of the rewards given to them. To enable this, two small wooden picture frames were hung on the chimpanzees’ cage mesh at the beginning of this Phase (Fig. 1). If the chimpanzee exchanged his/her token through one of the picture frames, s/he would then receive the high-value food reward (a grape) for completing the exchange, irrespective of the experimental condition. Prior to testing, the chimpanzees had all completed ‘Picture Frame Pre-exposure Sessions’, through which they had learned the contingency of exchanging a token through a picture frame (procedure described below). The Test Phase thus allowed us to examine whether the chimpanzees only chose to exchange for grapes in certain conditions (e.g., in response to inequity) or whether they universally attempted to obtain the best reward possible.

### Experimental conditions

The Different condition – in which the subject always received a less-preferred reward than their partner – created inequity, while the Low-value Same condition – in which both chimpanzees received the same low-value reward for every exchange – tested whether the chimpanzees’ responses were simply dictated by receiving a low-value reward, regardless of what their partner received. In the Unavailable condition, before each chimpanzee was offered a token to exchange, the experimenter first showed them a grape in order to highlight these high-value rewards in the environment but, after the chimpanzee exchanged, they were offered a piece of celery. This condition controlled for influences of individual contrast, which may also explain the chimpanzees’ responses in the Different condition (i.e., to determine whether there was a social component to any reward comparisons they made). Finally, in the High-value Same condition, both chimpanzees received grapes for each exchange. Therefore, in the Test Phase of this condition, the chimpanzees could not increase the quality of the reward they could receive; they were already receiving the maximum quality reward (a grape) for each exchange. This condition tested whether the chimpanzees exchanged tokens through the picture frame in a targeted manner, in order to maximize their rewards (either in relation to their partner’s rewards or to their own), or whether they did so whenever the picture frame was hung on their cage at the beginning of the Test Phase regardless of the condition (and what rewards they, and their partner, received in the Baseline Phase).

### Picture frame pre-exposure sessions

Prior to being tested in the experimental conditions, each chimpanzee experienced a series of pre-training trials to provide them with the causal information about the picture frames. These picture frames were the mechanism the chimpanzees could use in the Test Phase to improve the quality of the rewards they received. For these pre-exposure sessions, the chimpanzees were tested individually, rather than in a pair, and each chimpanzee received five sessions, each run on separate days.

For the first pre-exposure session, the chimpanzees had to make ten exchanges with the experimenter (Baseline Phase) and for every completed exchange, they were offered a piece of celery (the low-value reward). Once they completed this, a picture frame was hung on the cage mesh. The picture frame was wooden and the inside aperture of the frame was 10 cm^2^, such that it perfectly framed one hole of the cage mesh; in this way, the picture frame ‘highlighted’ a particular hole through which the chimpanzees could exchange a token (Fig. 1). The chimpanzees were then given the opportunity to make 10 more exchanges (Test Phase). In this first session, the chimpanzees were not trained, nor encouraged, to exchange the token through the picture frame. This was done to determine whether the chimpanzees would spontaneously exchange a token through the picture frame; being a novel object, the chimpanzees may have been interested to explore the frame purely because of its novelty. If the chimpanzee spontaneously exchanged a token through the picture frame they would have received a grape, but otherwise they would continue to receive a piece of celery for each exchange.

Of the 16 chimpanzees tested, only three spontaneously used the frame in their first pre-exposure session. Of these three, one female exchanged the token through the picture frame only once (despite having received a grape, she never repeated the behavior), while, of the two males that used the picture frame, one exchanged the token through the picture frame for all of the 10 possible trials and the other used the picture frame for five trials after trying out this new technique on his sixth attempt (i.e., after discovering the properties of the picture frame, he exchanged all his tokens through it to get the high-value reward).

All 16 chimpanzees were tested in four more pre-exposure trials which included 10 exchanges in a Baseline Phase followed by 10 exchanges in a Test Phase as described above. For the second pre-exposure trial, the chimpanzees received a grape for every exchange (High-value Same condition reward schedule), regardless of whether they exchanged their token through the picture frame or not in the Test Phase. In the third pre-exposure session the chimpanzees were rewarded with a piece of celery for every exchange in the Baseline Phase and thus, in the Test Phase, were again exposed to the significance of the picture frame. Again, we did not want to train the chimpanzees to exchange tokens through the frame, as we did not want exchanging through the picture frames to become a ‘learned trick’ that the chimpanzees did on cue simply whenever they saw the picture frame. Rather, the experimenter ‘showed’ the chimpanzee how to use the picture frame by holding her hand at the bottom of the aperture created by the picture frame so that, as the chimpanzee returned the token to her hand, it did so by returning the token through the picture frame. This procedure was repeated until the chimpanzee spontaneously exchanged their token in this manner. The chimpanzee was then rewarded with a grape rather than the piece of celery for each exchange made through the picture frame. Other than this conditioning, no overt training technique or secondary reinforcer (“bridge”, e.g., a clicker) was used. For the fourth pre-exposure session, as for the second session, the chimpanzees received a grape for every exchange in both the Baseline and Test Phases. This session was run to demonstrate to the chimpanzees that, if they used the picture frame in a condition in which they were already receiving a grape for each exchange, their reward value stayed constant (i.e., they received the high value reward whether they exchanged the token through the picture frame or not).

The final, fifth, session again followed the Low-value Same reward schedule but, in this, the experimenter placed her hands on her knees after the chimpanzee took the token so as not to actively cue the chimpanzee to exchange their token through the picture frame in the Test Phase. Thus for this session the chimpanzees made 10 exchanges for which they received a piece of celery (Baseline Phase), the picture frame was hung on their cage mesh, and they could then make a further 10 exchanges for which they could improve their rewards by exchanging through the picture frame (Test Phase).

We determined that a chimpanzee understood the contingency of exchanging a token through the picture frame if they did so for a minimum of 8/10 exchanges in this fifth pre-exposure session. All 16 chimpanzees understood this contingency; 10 made 100% of their exchanges through the picture frame in this fifth session, three exchanged 9 tokens through the picture frame, and three exchanged 8 tokens through the picture frame. There was no difference in the responses shown by males or females. Given the chimpanzees’ responses in this fifth pre-exposure session, we considered that all 16 chimpanzees recognized the properties of the picture frame. After both chimpanzees in a test-pair completed all five pre-exposure sessions, and were considered to understand the contingency of exchanging through the picture frame, they commenced with the testing schedule for the experimental conditions. As all pre-exposure sessions were conducted on consecutive days through the week starting on Monday, there was always a two-day break between the final pre-exposure session (Friday) and the first experimental session (Monday).

### Procedure

For every experimental test session, the pairs of chimpanzees were called in to one of their inside dens of their home cage by the experimenter. These inside dens were part of their typical housing, and thus were a familiar area for the chimpanzees. Only those chimpanzees that came in voluntarily were tested, but because of the chimpanzees’ familiarity with the experimenter, and their positive relationship with her, the chimpanzees chose to participate in testing on an almost daily basis. If a chimpanzee did not come in, the experimenter tried again the next day. No pair was ever tested more than once a day and there was never more than a three-day period between test sessions (no testing occurred over the weekend). After calling both chimpanzees into the inside testing den, the experimenter placed two food containers on the floor directly in front of the chimpanzees. One contained grapes and the other contained pieces of celery. Both food rewards could easily be seen by the chimpanzees and both food rewards were on display in all conditions. The positioning of these containers was counterbalanced across conditions so that neither their presence, nor their relative position, would cue the chimpanzees’ responses.

In all four conditions, the chimpanzees were tested first in the Baseline Phase and then in the Test Phase to first expose them to the quality of rewards that both the subject and partner received (Baseline Phase) and then to test how the Subjects responded when given the opportunity to change the quality of rewards they received (Test Phase). After completing the 20 trials in the Baseline Phase (10 exchanges per chimpanzees), the chimpanzees were immediately tested in the Test Phase. For the Test Phase, the experimenter produced two previously-hidden wooden picture frames and attached them to the chimpanzees’ caging (Fig. 1). There was no temporal gap between the two Phases, apart from the time it took for the experimenter to quickly hang the two picture frames on the caging (they could be hung easily and quickly with double-ended spring clips). Two picture frames were hung on the mesh so that one chimpanzee could not monopolize access to a single frame. The distance between the two picture frames when they were hanging on the cage front was roughly 1.5 m. In the Test Phase, if either chimpanzee exchanged their token through one of the picture frames, they would receive the high-value food reward (a grape) irrespective of the condition. Exchanging the tokens in this manner would require some cognitive effort on the part of the chimpanzees (in order to select a specific hole through which to exchange tokens) but would not have required any additional physical effort as the action of exchanging a token was the same. In the Test Phase, as in the Baseline Phase, the chimpanzees took turns to each make ten exchanges with the experimenter (20 trials in total).

In both Phases, Baseline and Test, the experimenter started with the partner chimpanzee and alternated between the two, offering a token and then giving them a reward if they exchanged. Every time the experimenter offered a chimpanzee their food reward, she held it up in front of both chimpanzees so that both could see the reward. Specific to the Test Phase, the experimenter squatted down in between the two picture frames and so was central to the cage front. For every exchange, the experimenter offered the chimpanzee the token directly in between the two picture frames so as not to cue the chimpanzee to exchange in a particular location. Furthermore, the moment that the chimpanzee took the token from the experimenter, the experimenter laid her hands on her knees. In this way, she was not “asking” for the token and did not show the chimpanzee where on the cage the token “should” be exchanged. As soon as the chimpanzee had pushed more than 50% of the token back through the cage (either through the picture frame or another hole in the mesh) the experimenter then reached up and took the token (Fig. 1).

### Coding and analysis

For every completed exchange, the chimpanzee was offered a food reward. If the chimpanzees failed to exchange the token or did not accept the reward offered to them, this was classed as a “refusal”. A chimpanzee could refuse to accept the token within 10 s or they could take it but not return it within 30 s. These actions were both coded as token refusals. Food-reward refusals were similar; they were coded when an animal did not accept the food item within 10 s, took the food item but did not eat it within 30 s, or took the food but then pushed it back outside their cage uneaten. The experimenter recorded every response that the chimpanzees made (exchanges and refusals) in real-time during the experiment. The experimenter also recorded the latency for the chimpanzee to exchange the token back to the experimenter (note that any exchanges in excess of 30 s were classed as refusals). All test sessions were video-taped.

Generalized Linear Mixed Models (GLMM) were used to compare the responses of the chimpanzees across conditions, in which “pair” was a fixed effect. There was no effect of the pair in which chimpanzees were tested on the proportion of exchanges made through the picture frames in the Test Phase (*t* = −0.24, df = 14, *P* = 0.811). The same was also true (i.e., that there was no effect of ‘pair’) when considering the number of refused exchanges in either the Baseline Phase (*t* = 1.81, df = 14, *P* = 0.257) or the Test Phase (*t* = 1.04, df = 14, *P* = 0.318). To determine whether the chimpanzees’ behavior varied between conditions and Phases, we conducted nonparametric Wilcoxon signed-ranks tests for related samples, and to compare the responses of male and female chimpanzees, we used a Mann Whitney U test for unrelated samples. As multiple pair-wise comparisons were conducted, to account for familywise errors, we applied a Holm’s sequential Bonferroni method (Holm, 1979). All analyses were conducted in SPSS 20 (IBM) and HLM7 Student (Scientific Software International). Graphs were produced in R (R Core Team, 2012) using ggplot2 (Wickham, 2009). All tests were two-tailed.

## Results

### Did attempts to get high-value rewards vary by condition?

**Figure 2:**
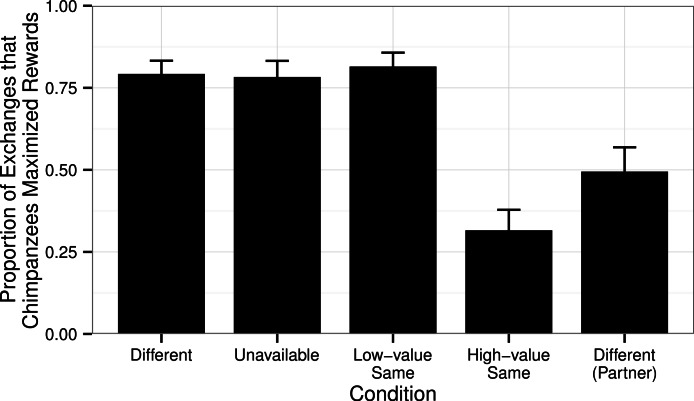
Proportion of exchanges through the picture frames in the Test Phase. The proportion of the chimpanzees’ exchanges in which the chimpanzees returned the token through the picture frame in the Test Phase in each of the four conditions (when tested as the subject) in order to obtain the high-value reward (a grape). Also shown are the responses of the chimpanzees in the Different condition when tested in the role of the partner (for which, like subject chimpanzees in the High-value Same condition, they received a high-value grape regardless of whether they exchanged their tokens directly through the mesh, or through the picture frame, Table 1).

In the Test Phase, chimpanzees could obtain a high-value reward if they exchanged a token through the picture frame. There was a significant difference in the proportion of trials in which the chimpanzees exchanged tokens through the picture frame across conditions in this Test Phase (GLMM Estimate of Fixed Effects: *t* = −4.89, df = 56, *P* < 0.001, Fig. 2). In conditions in which the chimpanzees received a less-preferred reward (irrespective of what their partner received), they took advantage of the opportunity provided to them (the picture frames), exchanging through the picture frame significantly less often in the High-value Same condition than in the Different (Wilcoxon’s signed-ranks test, *T* + = 9.5, *N* = 32, *P* < 0.001), Unavailable (*T* + = 373.5, *N* = 30, *P* < 0.001), and Low-value Same (*T* + = 485.5, *N* = 32, *P* < 0.001) conditions.

Considering the Different condition, chimpanzees were more likely to use the picture frame in the Test Phase when they were the subject (and could improve their rewards) than when they were tested as the partner (and already received high-value rewards: Wilcoxon’s signed-ranks test, *T* + = 319.0, *N* = 32, *P* < 0.001). Intriguingly, despite receiving the high-value reward for all exchanges in the Baseline Phase, in the Test Phase subjects used the picture frame more in the Different condition than in the High-value Same condition (*T* + = 55.5, *N* = 32, *P* = 0.021, Fig. 2), most likely induced by social facilitation arising from them seeing their test partner exchanging tokens through the picture frame.

### Refusals: the baseline and test phases compared

There was a significant difference in the number of refusals made by chimpanzees across conditions in the Baseline Phase (GLMM Estimate of Fixed Effects: *t* = −4.69, df = 56, *P* < 0.001), but there was no such difference in the Test Phase (*t* = −1.41, df = 56, *P* = 0.164).

**Figure 3:**
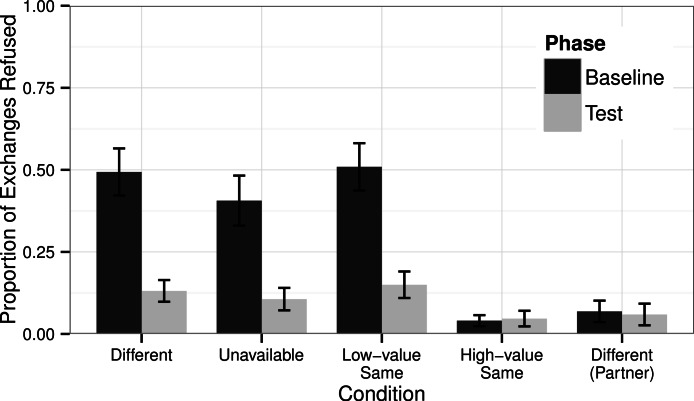
Proportion of Refusals. The proportion of trials in which chimpanzees refused: either to exchange or to eat the offered reward. Black bars show the chimpanzees’ responses in the Baseline Phase when they had no opportunity to improve their rewards and gray bars show their responses in the Test Phase when they could obtain a high-value grape if they exchanged their token through the picture frame. Also shown are the responses by the chimpanzees when tested in the role of the partner in the Different condition for which their pay-off structure was identical to chimpanzees tested in the High-value Same condition (Table 1).

In those conditions in which the subject received less-preferred rewards in the Baseline Phase (irrespective of what their partner received), they refused less in the Test Phase when they were afforded the opportunity to obtain high-value rewards (Fig. 3). This was true in the Different condition (Wilcoxon’s signed-ranks test, *T* + = 7.0, *N* = 32, *P* < 0.001), the Unavailable condition (*T* + = 8.0, *N* = 32, *P* = 0.001), and the Low-value Same condition (*T* + = 12.5, *N* = 32, *P* < 0.001). Subjects showed no difference in the number of refusals they made in the High-value Same condition in the Baseline Phase compared to the Test Phase (*T* + = 140.5, *N* = 32, *P* = 0.939).

When tested in the role of the partner in the Different condition the chimpanzees received a high-value reward for every exchange. This reflected the reward pay-outs for chimpanzees tested as the subject in the High-value Same condition. In neither situation should the chimpanzees exchange tokens through the picture frame in the Test Phase as they were already being given the maximum value reward for each exchange. To confirm this, we compared the responses of chimpanzees in the role of the partner in the Different condition when in the Baseline Phase compared to the Test Phase. We determined no differences in their refusals across Phases (*T* + = 18.0, *N* = 32, *P* = 0.584). This mirrors the responses of the subjects in the High-value Same condition in the Baseline compared to the Test Phase.

### Exchange latencies

For each of the four conditions, there was no difference in the time it took chimpanzees to exchange a token in the Baseline Phase compared to the Test Phase: Different (Wilcoxon’s signed-ranks test, *T* + = 343.0, *N* = 31, *P* = 0.063), Unavailable (*T* + = 210.0, *N* = 28, *P* = 0.873), Low-value Same (*T* + = 227.5, *N* = 30, *P* = 0.918), and the High-value Same (*T* + = 203.5, *N* = 32, *P* = 0.383). In the Baseline Phase there was no difference in the time it took them to return the token to the experimenter across the four conditions (Friedman’s test: *X*^2^(3) = 1.978, *N* = 27, *P* = 0.577) however, there was a difference in the exchange latencies across conditions in the Test Phase (*X*^2^(3) = 10.333, *N* = 27, *P* = 0.016, Table 2). In the Test Phase, subjects returned the token more quickly in the High-value Same condition – when both chimpanzees were given grapes for their exchanges – compared to those conditions which created either inequity (Different condition: *T* + = 113.0, *N* = 31, *P* = 0.008) or individual contrast (Unavailable condition: *T* + = 80.5, *N* = 28, *P* = 0.005). There was, however, no difference between the subjects’ exchange latencies in the High-value Same compared to the Low-value Same condition, when the Holm-Bonferroni correction was applied (*T* + = 341.0, *N* = 30, *P* = 0.026, Table 2). There was no difference in the time it took chimpanzees to return the tokens across all those conditions in which the subjects had received the low-value celery pieces for every exchange, regardless of what they, or their partner, had been previously offered (i.e., comparing the Different, Unavailable, and Low-value Same conditions: (*X*^2^(2) = 3.63, *N* = 27, *P* = 0.163, Table 2).

**Table 2:** The average duration of exchanges. The average time it took chimpanzees to exchange tokens with the experimenter in each of the four conditions across both the Baseline Phase and the Test Phase. Across all conditions, in the Baseline Phase, the average latency for a chimpanzee to return a token was 2.55 s (range: 0.91–10.65 s) while in the Test Phase the average exchange latency was 2.41 s (range: 0.92–8.94).

	Average exchange latency (range)/seconds	
	Phase A	Phase B
**Different**	**2.34** (0.91–5.84)	**2.72** (1.15–8.94)
**Unavailable**	**2.68** (0.96–6.03)	**2.43** (1.14–5.41)
**Low-value Same**	**2.69** (1.16–10.65)	**2.51** (1.08–6.08)
**High-value Same**	**2.53** (0.92–4.79)	**2.00** (0.92–4.79)

## Discussion

In the context of this experimental test, chimpanzees responded in a targeted manner in order to obtain better quality food items. Importantly, the chimpanzees’ responses appeared to be context-dependent; they only exchanged tokens through the picture frame when they had the possibility of increasing their reward values. We note that, although the chimpanzees had to determine whether to exchange a token through the picture frame in the Test Phase, doing so did not require additional physical effort. Therefore, the chimpanzees’ context-specific responses are unlikely explained by the extra effort required, but rather because they only attempted to improve their rewards when there was the option for them to do so (i.e., not in the High-value Same condition when they already received the highest quality reward available). Furthermore, we found that the chimpanzees participated more (i.e., they refused less) in those situations when they had the opportunity to receive a better reward than when they did not (i.e., in the Test Phase compared to the Baseline Phase). Overall, the chimpanzees’ responses appeared to be primarily influenced by the quality of the rewards they received, and not in relation to their partner’s rewards. However, the actions of their test partner did appear to influence the subjects’ motivation to exchange tokens through the picture frame, most likely driven by social facilitation (e.g., Hoppitt, Blackburn & Laland, 2007). We discuss the interplay of these individual and social factors in turn.

In the present study, the chimpanzees were equally likely to use the individual control provided by the picture frame whenever they received the less-preferred reward, irrespective of whether their partner received the same reward (Low-value Same condition) or a better one (Different condition). It is noteworthy, however, that the chimpanzees were not simply trained to exchange tokens through the picture frame in the Test Phase, but rather they responded in a targeted manner in order to better their rewards (when possible). Specifically, in those conditions in which the chimpanzees received the more-preferred, high-value reward, they were significantly less likely to use the picture frame in the Test Phase. The chimpanzees’ responses, both within a condition (when tested as the subject *versus* as the partner in the Different condition) and across conditions (Low-value Same *versus* High-value Same, when tested as the subject), demonstrated that the chimpanzees were not conditioned to use the picture frame whenever it was offered to them. Rather, the chimpanzees did so selectively in the Test Phase, when they had received low-value rewards in the Baseline Phase.

Intriguingly, when tested in the role of the partner in the Different condition, despite receiving high-value rewards (grapes) for their exchanges, chimpanzees used the picture frame more often in the Test Phase than when they were tested as the subject in the High-value Same condition (for which they also received grapes for their exchanges). In neither circumstance would exchanging tokens through the picture frame in the Test Phase have provided benefit – they were already gaining the best reward possible for each exchange. We propose that the partners’ responses may have been driven by response facilitation, the already-known behavior of exchanging tokens through picture frames was elicited after observing a conspecific performing the same act (see also Huber et al., 2009; Bates & Byrne, 2010; Hopper et al., 2013). In the Different condition, chimpanzees in the role of the partner saw the subject they were tested with use the picture frame in the Test Phase to improve the quality of their rewards. Observation of this may have caused the partner to also more often exchange tokens through the picture frame.

Ultimately, the chimpanzees were motivated to maximize their gains, regardless of how their rewards compared to those received by their partner (i.e., not in response to inequity, Brosnan et al., 2010) or to what rewards were available in the environment (i.e., not in response to individual contrast, Bräuer, Call & Tomasello, 2009). However, we note that in this study, in contrast to previous tests of inequity (e.g., Bräuer, Call & Tomasello, 2009; Brosnan et al., 2010), the chimpanzees had the ability to alter the value of the rewards they received in the Test Phase (in a typical test of inequity the subject’s responses do not impact the rewards they are given). Even though the chimpanzees’ responses did not reflect a behavioral response to inequity, their responses do reflect those reported previously for this species in tests of prosocial behavior. For example, Jensen et al. (2006) concluded from their test of chimpanzee altruism that “chimpanzees made their choices based solely on personal gain” (p. 1013; see also Silk et al., 2005; Vonk et al., 2008, but see Horner et al., 2011). Comparably, in our study, the chimpanzees’ responses indicated a desire to receive the highest quality reward available, regardless of how it compared to what their partner received; the chimpanzees were interested in their own rewards, not those of others. Note, however, that in our test, and unlike that of Jensen et al. (2006), the chimpanzees’ actions could not influence what their partner received, only what they received themselves, so ours was not a test of prosocial behavior. That is, if the subject wanted to equalize outcomes, they had to forsake their own better outcome, while a better test of prosocial behavior would be whether the subject would take action to bring both themselves and their partner a better option (c.f. Horner et al., 2011).

The time it took for the chimpanzees to return tokens revealed that exchanging a token through the picture frames took longer than exchanging tokens directly through the cage mesh. In the Test Phase, the chimpanzees returned tokens to the experimenter significantly more quickly in the High-value Same condition compared to the Unavailable and Different conditions. This is most likely because, in the High-value Same condition, the chimpanzees used the picture frame far less than in other conditions and so their responses were, on average, quicker. We propose that an interesting ‘next step’ would be to investigate whether requiring the subjects to exert an even greater effort to increase their reward values would elicit the same responses. In this way, we could ask whether individuals would still attempt to improve their rewards if more effort was required and to determine whether their attempts to do so vary across conditions (for example, only when they experienced inequity but not frustration, or the reverse).

In conclusion, although the chimpanzees’ responses did not appear to reflect a response to inequity, or individual contrast, they did change their responses according to the experimental context. It has been proposed that captive chimpanzees, in such tests of cognition, may suffer from learned helplessness and accept whatever quality of reward is offered to them by a human experimenter (Visalberghi & Anderson, 2008). Given this, and the proposal that the learning ability of chimpanzees may be conservative, causing them to become “stuck” using a particular behavioral response (e.g., Hrubesch, Preuschoft & van Schaik, 2009; Marshall-Pescini & Whiten, 2008), it is promising that, in this study, the chimpanzees varied their responses. That the chimpanzees were able to alter their behavioral responses, depending on the condition, and in order to gain the highest value rewards possible, provides further support to recent examples of chimpanzees’ flexible learning (e.g., Manrique, Völter & Call, 2013; Yamamoto, Humle & Tanaka, 2013). Considering this study more broadly, we note that the simplicity of this design means that it could be easily adapted for use with other species and this represents a novel starting point for a comparative understanding of how animals respond to differential rewards when given the opportunity to alter those outcomes.
